# Effect of Nurse-Led Individualised Self-Care Model on Myocardial Infarction Patients with Diabetes: A Randomised Controlled Pilot Trial

**DOI:** 10.31083/j.rcm2401031

**Published:** 2023-01-16

**Authors:** Jia Zhang, Xue-Mei Zhou, Ke-Ke Qian, Jian-Dong Yu, Hong-Wei He, Li-Hua Zhao, Wen-Wen Yang, Gui-Ling Geng, Hong-Wu Shen

**Affiliations:** ^1^Department of Cardiology, Affiliated Hospital of Nantong University, 226000 Nantong, Jiangsu, China; ^2^School of Medicine, Nantong University, 226019 Nantong, Jiangsu, China

**Keywords:** myocardial infarction, diabetes, individualised self-care model

## Abstract

**Background:**

To assess the effectiveness of the nurse-led individualised self-care model on myocardial infarction (MI) patients with diabetes.

**Methods:**

A total of 120 MI patients were enrolled from May 2020 to December 2021. The intervention group received the nurse-led individualised self-care model (n = 60), whereas the control group only received routine health education (n = 60). The Myocardial Infarction Dimensional Assessment Scale (MIDAS), Coronary Heart Disease Self-Management Behavior Scale (CSMS), Self-Rated Abilities for Health Practices (SRAHP) scale, General Self-Efficacy Scale (GSES), Hospital Anxiety and Depression Scale (HADS), blood glucose and nursing satisfaction in both groups were observed and recorded.

**Results:**

The six MIDAS subscales except for insecurity, and all dimensions of the CSMS, SRAHP, GSES and HADS scores, of the intervention group were significantly improved compared to those of the control group (*p <* 0.05). Compared with the control group (5.69 ± 1.43 mmol/L), the intervention group showed a decrease in the serum levels of fasting blood glucose (4.83 ± 1.57 mmol/L; *p <* 0.01).

**Conclusions:**

Our pilot study provides preliminary evidence supporting the feasibility of implementing nurse-led individualised self-care, suggesting its preliminary effects in improving health-related quality of life, self-care ability, health behaviours, self-efficacy, social support and nursing satisfaction among MI patients with diabetes. However, considering the unblinded and pilot nature of this study, these positive results should be interpreted with caution.

**Clinical Trial Registration:**

OSF Registration number: DOI 10.17605/OSF.IO/DVW95 (https://archive.org/details/osf-registrations-dvw95-v1).

## 1. Introduction

Myocardial infarction (MI) is a serious cardiovascular disease that leads to severe and lasting acute myocardial ischemia due to the interruption of coronary blood flow. Around 1.55 million people die of cardiovascular diseases in developing countries every year, of which about half are caused by MI [1, 2]. In 2014, the MI mortality rate in China was 55.32/100,000 in urban areas and 68.6/100,000 in rural districts [3]. The high disability rate, high recurrence rate, and serious complications of MI make it bring a heavy social and family burden. The National Cholesterol Education Program expert panel in the US reported that long-term elevated blood glucose was not only a prominent independent risk factor for coronary atherosclerosis but also a risk factor for diabetes mellitus (DM) vascular disease and even MI [4]. A complex association exists between DM and MI. MI patients with diabetes often show specific metabolic, neuro-immune and structural cardiac injuries [4]. Previously, a meta-analysis of 698,782 patients in 102 cohort studies showed that the occurrence of DM was significantly positively correlated with the increased risk of cardiovascular diseases [5]. Moreover, a 9-year follow-up cohort study conducted by Abaci revealed that the incidence of MI in diabetic patients was significantly higher than that in non-diabetic patients (10.8% vs. 3.9%) [6]. In addition, a prospective study of 5934 patients with DM who had been followed up for 10 years indicated that the risk of cardiovascular events in patients with a diabetes duration greater than or equal to 12 years was comparable to that in patients with previous MI without diabetes [7]. Furthermore, diabetic patients have more angiopathies, and these mainly involve coronary artery lesions. For example, Granger *et al*. [8] showed that the incidence rate of coronary artery lesions was significantly higher in diabetic patients than in non-diabetic patients (66% vs. 46%).

Therefore, the European Society of Cardiology (ESC) suggests that the individual self-care model should be used for this comorbid group (MI patients with diabetes) in many aspects such as diet, medicine and health guidance to help them improve their quality of life and activity tolerance [9]. The nurse-led individualised self-care model is based on the theory of self-efficacy, which defines the cardiovascular nursing specialist as the leader of the team. In the nurse-led individualised self-care model, nurses and team members formulate achievable goals with patients according to the individual disease situation and adopt targeted individualised nursing programmes to promote changes in patients’ lifestyles to improve their health. The action mechanism of the model could be summarised as follows: nurses and team members should focus on the specific clinical characteristics of patients with chronic diseases, formulate personalised nursing plans and improve patients’ disease knowledge through diversified forms of health education. In the process of rehabilitation nursing, nursing specialists should encourage patients to actively participate in disease self-management, which reduces the dependence of patients with chronic diseases on the nursing specialists and health caregivers and promotes the improvement of their self-care ability [10]. In addition, during the application of the nurse-led individualised self-care model process, nursing specialists should care for patients’ negative emotions and strengthen their medical adherence [11, 12]. Additionally, the nurse-led individualised self-care model can improve patients’ self-care ability through peer education, which ultimately improves their quality of life [13, 14].

The nurse-led individualised self-care model has been applied to a variety of chronic diseases. Yin *et al*. [15] found that after the implementation of diet management combined with the nurse-led individualised self-care model, the Body Mass Index (BMI), Hemoglobin A1c (HbA1c) and Fasting Blood Glucose (FBG) in the observation group were significantly lower than those in the control group (*p *< 0.05). Therefore, diet management combined with the model can effectively reduce blood glucose. Sasso *et al*. [16] reported that good glycaemic control during acute coronary syndrome plays a cardioprotective effect. Tight glycaemic control can improve myocarditis and fibrosis and protect the ultrastructure of the heart by reducing the expression level of lipid peroxide, inhibiting transforming growth factor (TGF)-β activation and downregulating the levels of nuclear factor-k-gene binding / NOD-like receptor thermal protein domain associated protein 3 (NF-κB/NLRP3) [17, 18, 19]. In addition, Tian *et al*. [20] found that the individualised self-care model could effectively improve the Minnesota Living with Heart Failure Questionnaire (MLHFQ) score and self-care ability score (*p *< 0.05).

Most of the previous studies [11, 12] have explored the effect of the nurse-led individualised self-care model on MI patients with diabetes in terms of reducing the rehospitalisation rate, decreasing adverse events and improving patients’ knowledge level. However, few have concentrated on the patients’ quality of life, self-efficacy, self-management ability, health behaviour and so on. Moreover, previous clinical trials [11, 12] have rarely focused on chronic disease patients in the community. Therefore, this research aimed to explore the effects of the nurse-led individualised self-care model on quality of life, self-efficacy, self-management ability, health behaviour and blood glucose control for MI patients with diabetes.

## 2. Materials and Methods

### 2.1 Clinical Trial Registration

This clinical trial is registered in the open science framework (OSF) clinical trial registry (registration number: DOI 10.17605/OSF.IO/DVW95), Internet Archive link: https://archive.org/details/osf-registrations-dvw95-v1.

### 2.2 Ethics Statement

This randomised controlled pilot trial was carried out according to the Declaration of Helsinki (2013) and approved by the ethics committee of the Affiliated Hospital of Nantong University (2022-K098-01). All patients signed informed consent.

### 2.3 Inclusion and Exclusion Criteria

#### 2.3.1 Inclusion Criteria

(1) Patients over 21 years old hospitalised for MI in the last 3 months. (2) Patients undergoing percutaneous coronary intervention and diagnosed with type 2 DM. (3) Activities of Daily Living Scale score of over 60 [21] and meeting the physical activity plan criteria of the ESC. (4) Able to follow the guidance of the researchers and take a low-sodium and low-carbohydrate diet during the clinical trial. (5) Able to understand and independently complete relevant scale information and independently use electronic devices such as mobile phones and social media such as WeChat. (6) Not having joined the cardiac rehabilitation programmes of the Affiliated Hospital of Nantong University or other institutions before the clinical trial. (7) To avoid patients with MI who had previously received lifestyle intervention or symptom self-management programmes, this clinical study only included patients who had been diagnosed with MI for the first time.

#### 2.3.2 Exclusion Criteria

(1) Participants whose patient health questionnaire (PHQ)-9 scale score was over 10, given that depressive symptoms affect the process of the individualised self-care model. (2) Type 1 DM [22]. (3) Heart transplantation or renal failure requiring continuous renal replacement therapy (CRRT) at the time of enrolment. (4) Patients with acute exacerbations of chronic obstructive pulmonary disease (AECOPD) or previous stroke affecting physical functional activities. (5) Lack of communication tools, such as telephone or WeChat.

### 2.4 Statistics and Power Calculations

The sample size calculation was based on the self-efficacy score (mean = 2.88, standard deviation = 0.71, effect size = 0.82). The statistical significance level α was set to 0.05, the power (1-β) was set to 0.8 and the drop-out rate was set to 20% (a total of 120 participants were included according to the sample size calculation).

### 2.5 Participants, Randomisation and Blinding

Before commencing the sample recruitment, a blinded statistician used the SAS software version 9.2 (SAS Institute, Inc., Cary, NC, USA) package to generate two sets of non-duplicating random numbers for the two study groups. A team member who was not involved in recruitment or data collection prepared sealed opaque envelopes according to the generated randomisation list. After a participant was registered, a unique participant ID was assigned to them according to the sequence of their enrolment. The envelope was opened on site according to the participant’s ID, and the participant was randomised to the intervention group (*n* = 60) or the control group (*n* = 60) based on the randomisation list indicated by the intervention. The recruitment time for the two groups of participants was from May 2020 to December 2021. The recruitment location was the Department of Cardiology, Affiliated Hospital of Nantong University. Blinding the cardiovascular nursing specialists and participants was not possible, but the outcome assessors and statisticians were blinded.

### 2.6 Nursing Intervention

#### 2.6.1 Control Group

The patients in the control group received routine health education from cardiovascular nursing specialists. The frequency of routine health education was once a week for a total of 12 weeks. The content of the health education was strictly according to ESC guidelines [9] and the clinical diagnostic laboratory (CDL) program proposed by the The national cholesterol education program (NCEP). The specific implementations included self-monitoring and prevention of DM- and MI-related complications, exercise guidance, diet guidance and psychological counselling. At the end of each week’s intervention, the cardiovascular nursing specialist gave feedback on the last week’s intervention effect on MI patients with diabetes.

#### 2.6.2 Intervention Group

The intervention group received a 12-week nurse-led individualised self-care model intervention by cardiovascular nursing specialists. In the first stage (from 1 to 4 weeks), a pre-trained research nurse provided the MI–diabetes tool kit recommended by the American Heart Association (AHA) guidelines [10]. More details are summarised in the **Supplementary File**. The health education package adopted a combination of book and electronic materials. For patients with a low education level, one-on-one individual teaching was adopted by the nursing team. After the education and consultation meeting on the first day, the nurse-led individualised self-care model interventions were carried out for the first 4 weeks. Cardiovascular nursing specialists conducted telephone follow-ups twice a week and followed up home visits once every two weeks according to the individual situation of each patient and their family, which could give timely feedback on the situation of patients.

In the second stage (from 5 to 12 weeks), the cardiovascular nursing specialist strengthened the follow-up with the help of the WeChat platform and reviewed the self-recorded data of patients, including blood glucose and weight information. More detailed interventions are summarised as follows. (1) Diet and lifestyle adjustment: the cardiovascular nursing specialists emphasised a low-carbohydrate and low-salt diet, which could reduce inflammation of blood vessels. In addition, according to the tastes of patients, the cardiovascular nursing specialists encouraged them to eat more fresh fruits and vegetables to supplement their vitamins. In terms of lifestyle adjustment, smoking is a risk factor for poor prognosis of MI and DM. Therefore, the cardiovascular nursing specialists worked with patients to make a smoking cessation plan. (2) Medication guidance: the cardiovascular nursing specialists used pictures to teach patients about common adverse events of medicine and emphasised the importance of self-monitoring and regular medication, which could enhance adherence. (3) Anxiety and depression management: the cardiovascular nursing specialists recommended patients’ favourite ways of emotional relaxation, such as listening to light music, massage or deep breathing.

From week 8, patients returned to the Affiliated Hospital of Nantong University to receive special exercise training and consultation on exercise interventions. The cardiovascular nursing specialists strictly referred to the exercise guidelines of the ADA and AHA to recommend appropriate exercise interventions to patients. The recommended daily exercise time of patients was 30 to 40 minutes. In terms of aerobic exercise content, the cardiovascular nursing specialists recommended walking and gave information on a walking scheme. They used the intelligent cloud platform provided by the Affiliated Hospital of Nantong University to monitor the walking parameters of patients and timely updated and recorded the activity information of patients. They read the patients’ exercise information together with engineers and physiotherapists and adjusted the patients’ exercise intensity.

From the 10th week, the cardiovascular nursing specialist team invited the MI patients with diabetes to participate in the programme, which would provide social and peer support. The peer education leaders in this programme received 3-day project course training. The training content included disease knowledge of DM and MI and self-management training knowledge and skills. The training of peer educators was carried out by cardiovascular nursing specialists. Trained and qualified peer educators transformed their successful experience to MI patients with diabetes in the form of group lectures.

In the third stage (at week 13), the cardiovascular nursing specialists performed the last follow-up of MI patients with diabetes. The follow-up was conducted by WeChat video. The follow-up content included evaluation of disease status, self-management behaviour, health behaviour, quality of life, self-efficacy and nursing satisfaction.

### 2.7 Outcome Measures

#### 2.7.1 Primary Outcomes

(1) Health-related quality of life. This was evaluated by the Myocardial Infarction Dimensional Assessment Scale (MIDAS) [23]. In 2006, Wang *et al. * [24] translated this scale into Chinese and tested its reliability and validity. This scale includes 35 items and is divided into seven subscales: physical activity (12 items), security (nine items), emotional reaction (four items), dependency (three items), diet (three items), concerns over medication (two items) and side effects (two items). The Chinese version of the MIDAS had high reliability and validity (Cronbach’s α = 0.93). The questionnaire uses a 5-point Likert scoring system (0–4).

(2) Self-care ability. The Coronary Heart Disease Self-Management Behavior Scale (CSMS) was used to assess the self-care ability of MI patients with DM. The CSMS was developed by Ren *et al*. [25]. It contains 27 items in seven dimensions: lifestyle management (four items), general life management (four items), symptom management (four items), disease knowledge management (five items), first aid management (three items), medical adherence management (three items) and emotional cognition management (four items). Each item is scored by a 5-point Likert scoring system, with 1–5 points from ‘never’ to ‘always’.

(3) Health care behaviours. The Self-Rated Abilities for Health Practices (SRAHP) scale, developed by Becker *et al*. [26], was used to evaluate the health care behaviours of MI patients with diabetes. The Chinese version of the SRAHP was translated by Hu *et al*. [27]. This scale includes four dimensions (nutrition, psychological well-being, exercise and health responsibility), with a total of 28 items. The SRAHP adopts a 5-point Likert scoring system, in which ‘almost completely unsure’ is given 0 points, ‘a little confident’ is given 1 point, ‘medium confident’ is given 2 points, ‘relatively confident’ is given 3 points and ‘absolutely confident’ is given 4 points.

#### 2.7.2 Secondary Outcomes

(1) Self-efficacy. The General Self-Efficacy Scale (GSES) was developed by Schwarzer *et al*. [28]. Zhang *et al*. [29] translated and adapted it into Chinese. The Chinese version of the GSES has good reliability and validity, and its consistency coefficient is 0.75. The GSES has 10 items, scored on a 4-point system. The total score is between 10 points and 40 points.

(2) Social support. The Social Support Rate Scale (SSRS) was developed by Xiao* et al*. in 1994 [30]. This scale had good reliability and validity. It has 10 items and three dimensions, where a high score means high social support.

(3) Psychological status evaluation. The HADS was applied to assess AS-related psychological status before and after nursing interventions [31]. The Hospital Anxiety and Depression Scale (HADS) includes two dimensions: anxiety and depression. The score of each dimension is 0–21. A lower HADS score means better psychological status of AS patients.

(4) Laboratory examinations. The changes in HbA1c and FBG were compared between the two groups.

(5) Nursing satisfaction. The Newcastle Nursing Satisfaction Scale (NSNS) was used for assessing the nursing satisfaction of patients in both groups. It is divided into three grades: great satisfaction (>90 points), satisfaction (57–90 points) and unsatisfaction (<57 points).

### 2.8 Statistical Analysis

SPSS 20.0 statistical software (SPSS Inc., Chicago, IL, USA) was used for data analysis. The categorical variables (e.g., living, education level, gender, marital status, smoking, healthcare insurance, alcohol, nursing satisfaction) used the χ^2^ test. The independent *t*-test was performed for continuous variables (e.g., age, MIDAS, CSMS, SRAHP, GSES, SSRS, HADS). A value of *p *< 0.05 (two-tailed) indicated statistical significance.

## 3. Results

**Fig. 1.**
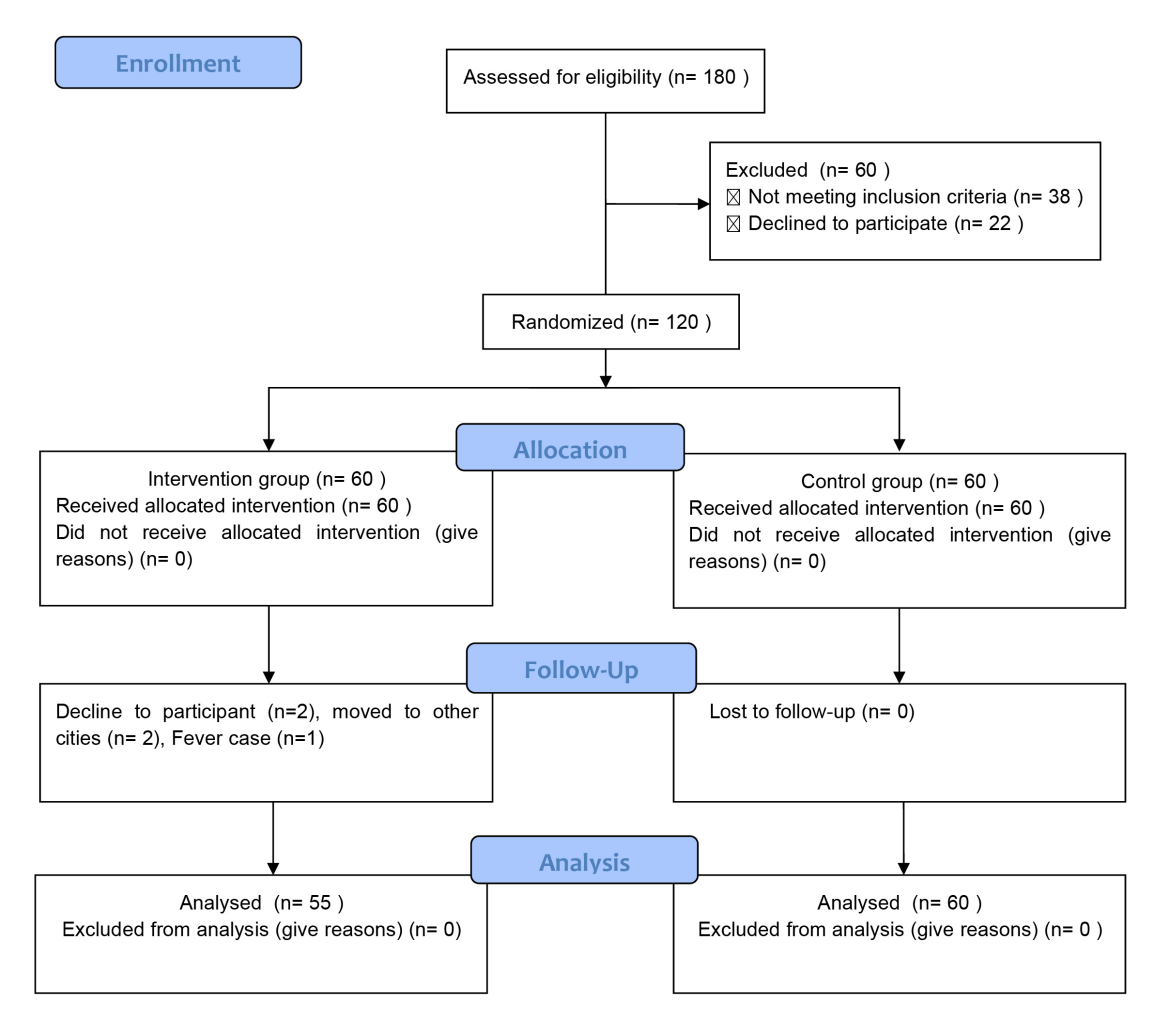
**Consort flowchart**.

From May 2020 to Dec 2021, 180 participants were screened in this study, and 65 were excluded [not meeting inclusion criteria (*n* = 38), declined to participate (*n* = 22), declined to participate in the intervention group (*n* = 2), moved to other cities in the intervention group (*n* = 2), fever case in the intervention group (*n* = 1)]. Finally, 115 participants were included for data analysis (Fig. 1).

### 3.1 Baseline Data

No difference in age, living, education level, gender, marital status, smoking, healthcare insurance, BMI or alcohol was observed between the two groups (*p *> 0.05; Table 1).

**Table 1.** **Baseline of study characteristics**.

		Intervention Group	Control Group	*t*/χ^2^ value	*p* value
		(*n = 55*)	(*n = 60*)		
Age (years)		67.31 ± 2.58	68.09 ± 2.77	1.56	0.12
Living:				0.08	0.78
	Urban	17	20		
	Rural District	38	40		
Education level:				0.14	0.71
	Undergraduate	1	2		
	High School	12	14		
	Middle School	42	44		
Gender, *n* (%)				0.01	0.92
	Male	39	42		
	Female	16	18		
Marital status:				0.05	0.83
	Married	51	55		
	Single	4	5		
Smoking:				0.23	0.63
	Yes	41	47		
	No	14	13		
Healthcare insurance:				0.78	0.38
	Yes	27	31		
	No	18	14		
BMI (kg/${\mathrm{cm}}^{2}$)		26.22 ± 1.67	26.78 ± 2.31	1.48	0.14
Alcohol:				0.01	0.93
	Yes	39	43		
	No	16	17		

### 3.2 Comparison of Health-Related Quality of Life between the Two Groups

**Fig. 2.**
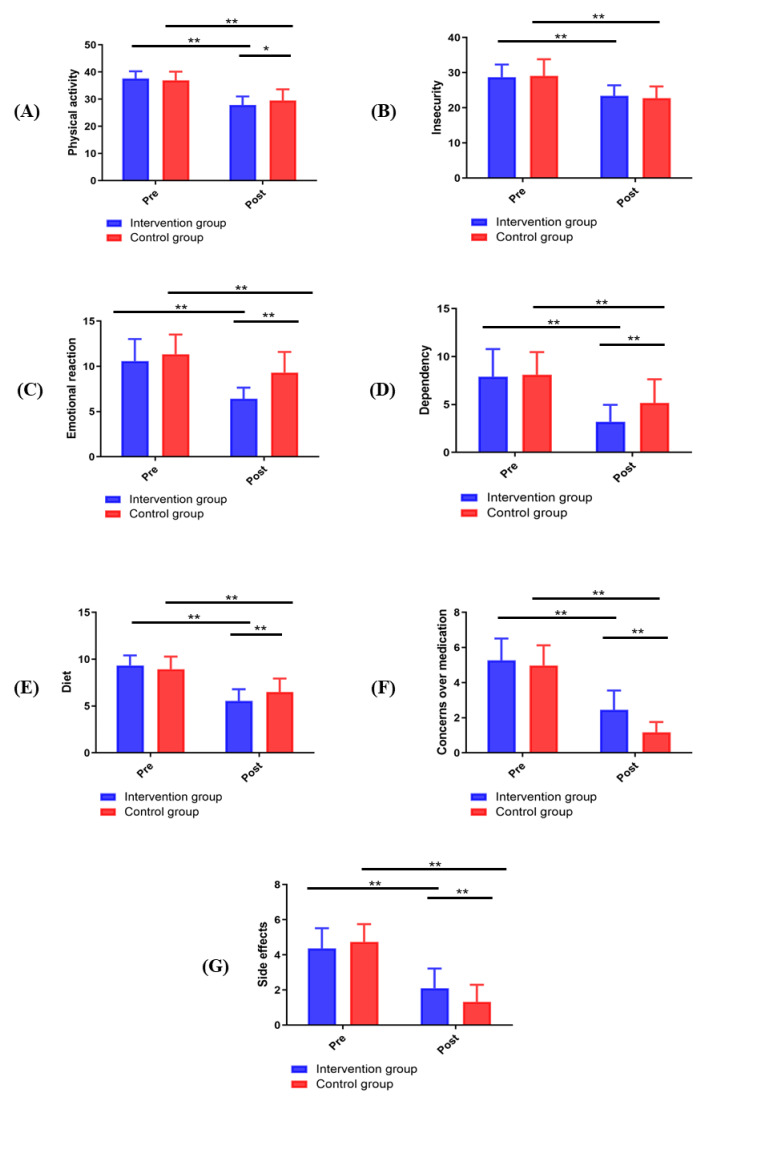
**Changes in MIDAS scores between groups**. (A) Physical activity. (B) Insecurity. (C) Emotional reaction. (D) Dependency. (E) Diet. (F) Concerns over medication. (G) Side effects. *p < 0.05, **p < 0.01.

The health-related quality of life measured by the MIDAS score was not statistically significantly different between the intervention group and the control group before the intervention (*p *> 0.05). After the 12-week intervention, except for the insecurity subscale, the other six subscale scores of the intervention group were significantly improved compared to those of the control group (*p *< 0.05; Fig. 2).

### 3.3 Comparison of Self-Care Ability between the Two Groups

**Fig. 3.**
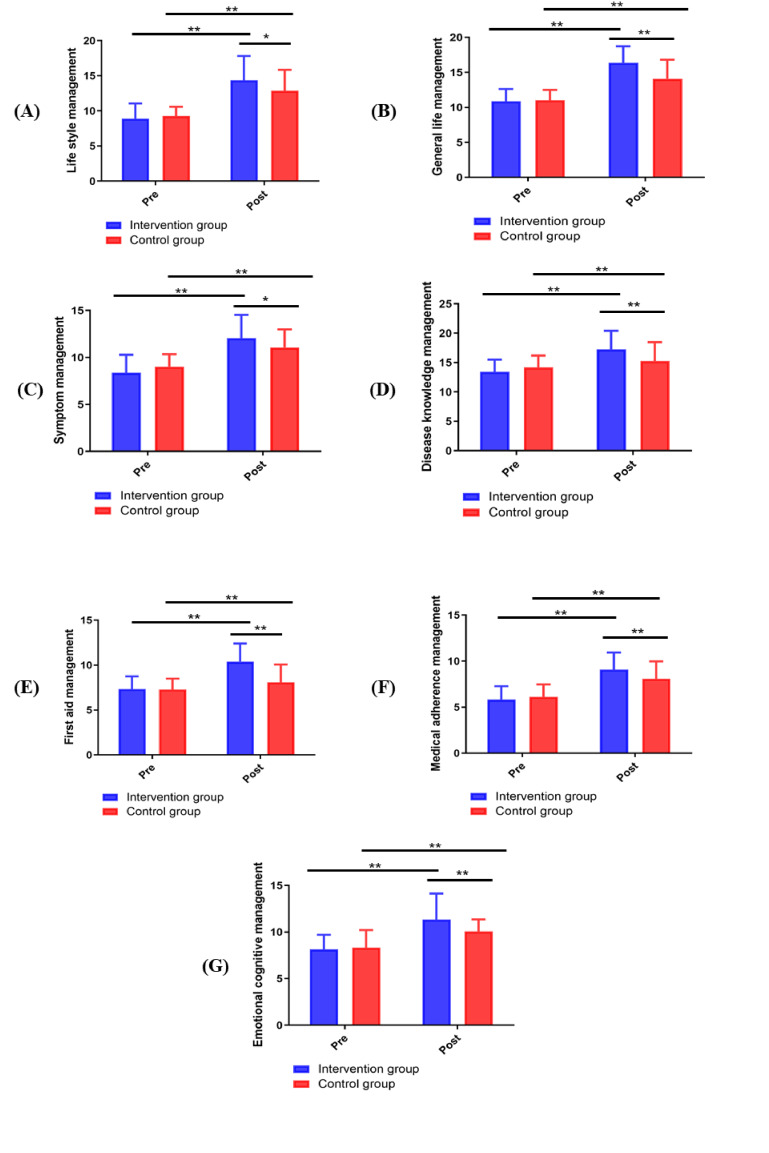
**Changes in CSMS scores between groups**. (A) Life style management. (B) General life management. (C) Symptom management. (D) Disease knowledge management. (E) First aid management. (F) Medical adherence management. (G) Emotional cognitive management. *p < 0.05, **p < 0.01.

The self-care ability measured by CSMS scores (lifestyle management dimension, general life management dimension, symptom management dimension, disease knowledge management dimension, first aid management dimension, medical adherence management dimension and emotional cognition management dimension) was not statistically significantly different between the intervention group and the control group before the intervention (*p *> 0.05). After the 12-week intervention, all dimensions of the CSMS score of the intervention group were significantly greater than those of the control group (*p *< 0.05; Fig. 3).

### 3.4 Comparison of Health Care Behaviours between the Two Groups

**Fig. 4.**
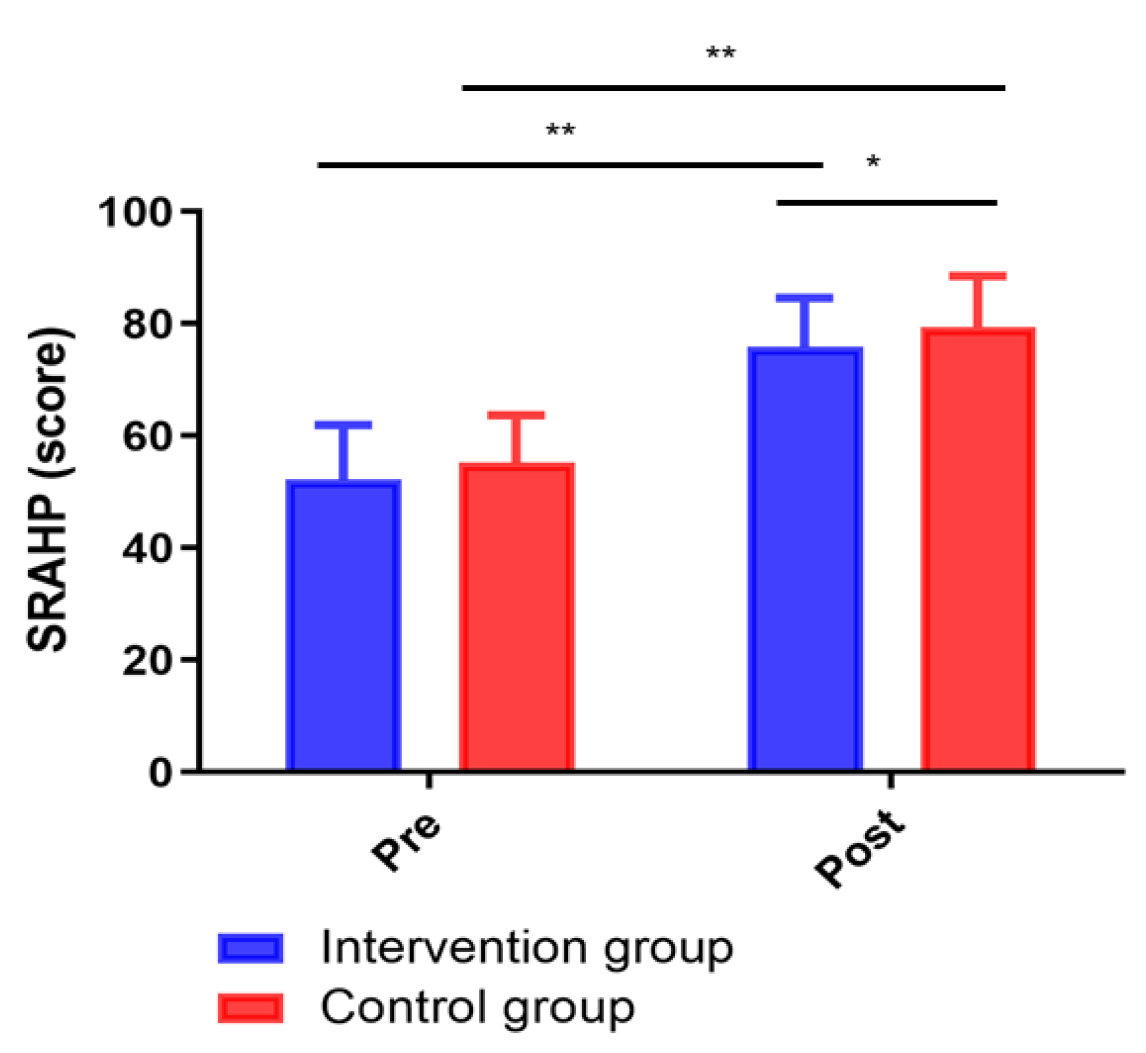
**Changes in SRAHP scores between groups**. *p < 0.05, **p < 0.01.

The health care behaviour measured by the SRAHP score was not statistically significantly different between the intervention group and the control group before the intervention (*p *> 0.05). After the 12-week intervention, the SRAHP score of the intervention group was significantly increased compared to that of the control group (*p *< 0.05; Fig. 4).

### 3.5 Comparison of Self-Efficacy between the Two Groups

**Fig. 5.**
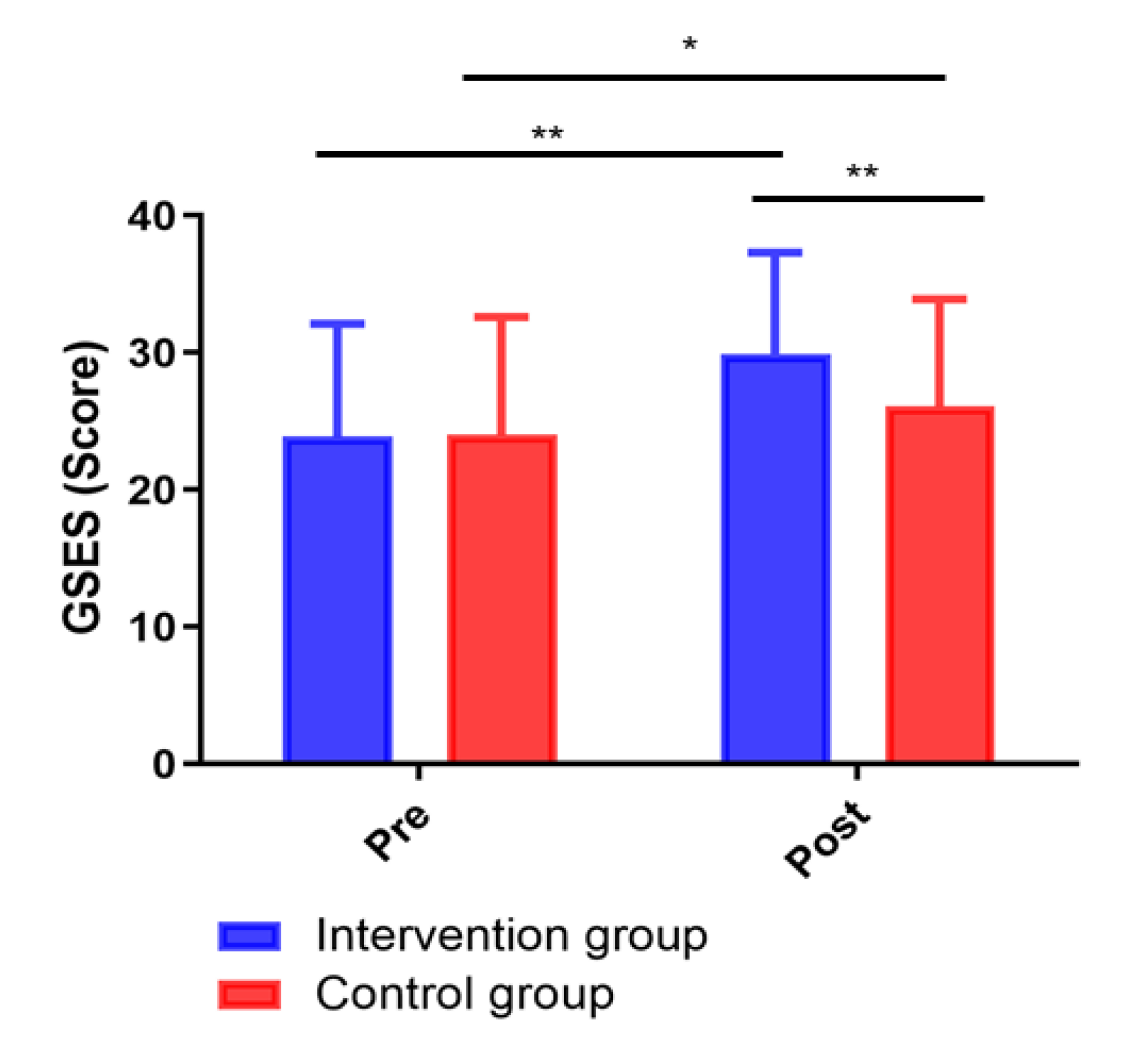
**Changes in GSES scores between groups**. *p < 0.05, **p < 0.01.

The self-efficacy measured by GSES score was not statistically significantly different between the intervention group and the control group before the intervention (*p *> 0.05). After the 12-week intervention, the GSES score of the intervention group was significantly increased compared to that of the control group (*p *< 0.05; Fig. 5).

### 3.6 Comparison of Social Support between the Two Groups

**Fig. 6.**
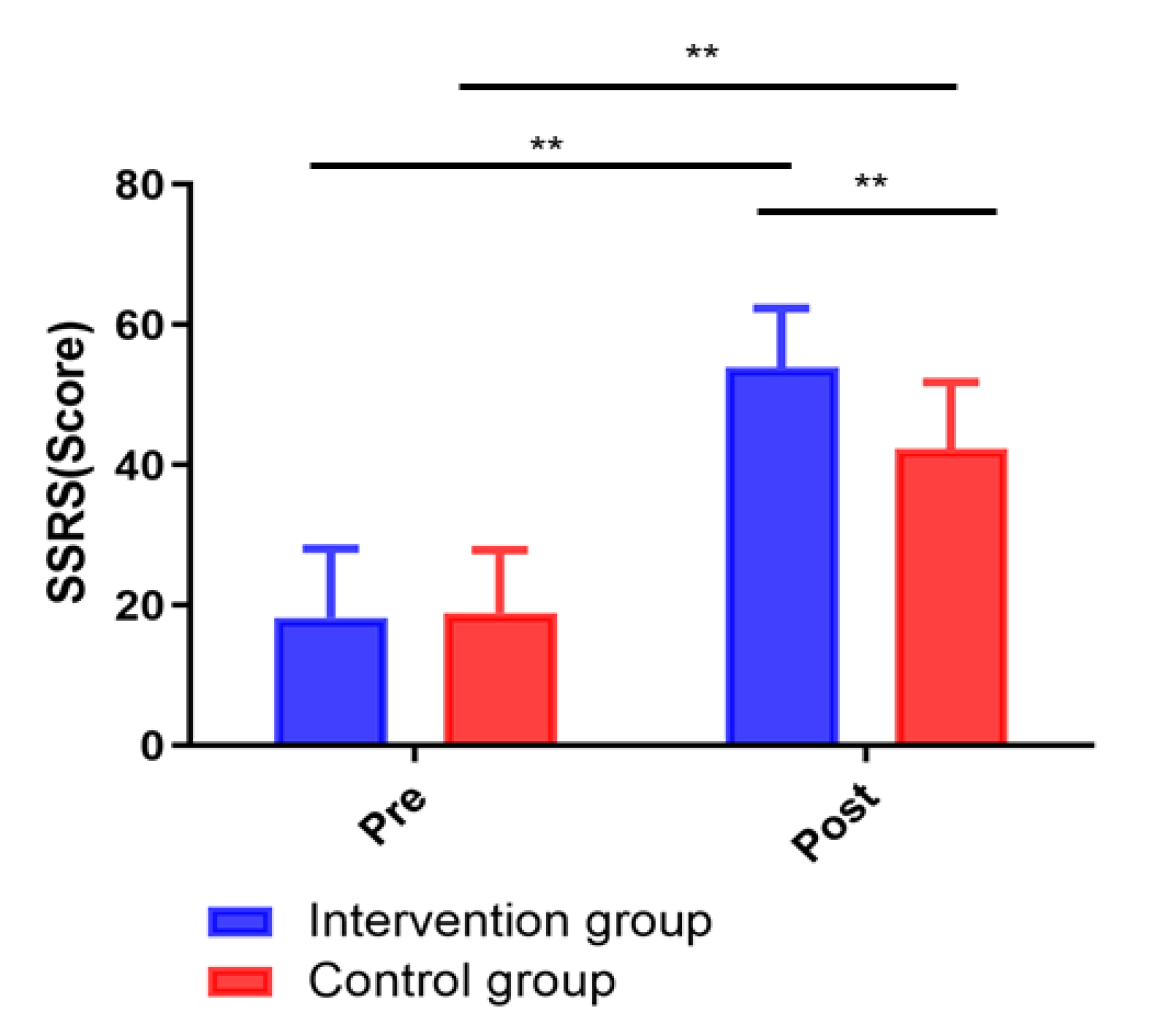
**Changes in SSRS scores between groups**. **p < 0.01.

The social support measured by SSRS score was not statistically significantly different between the intervention group and the control group before the intervention (*p *> 0.05). After the 12-week intervention, the SSRS score of the intervention group was significantly increased compared to that of the control group (*p *< 0.05; Fig. 6).

### 3.7 Comparison of Psychological Status between the Two Groups

**Fig. 7.**
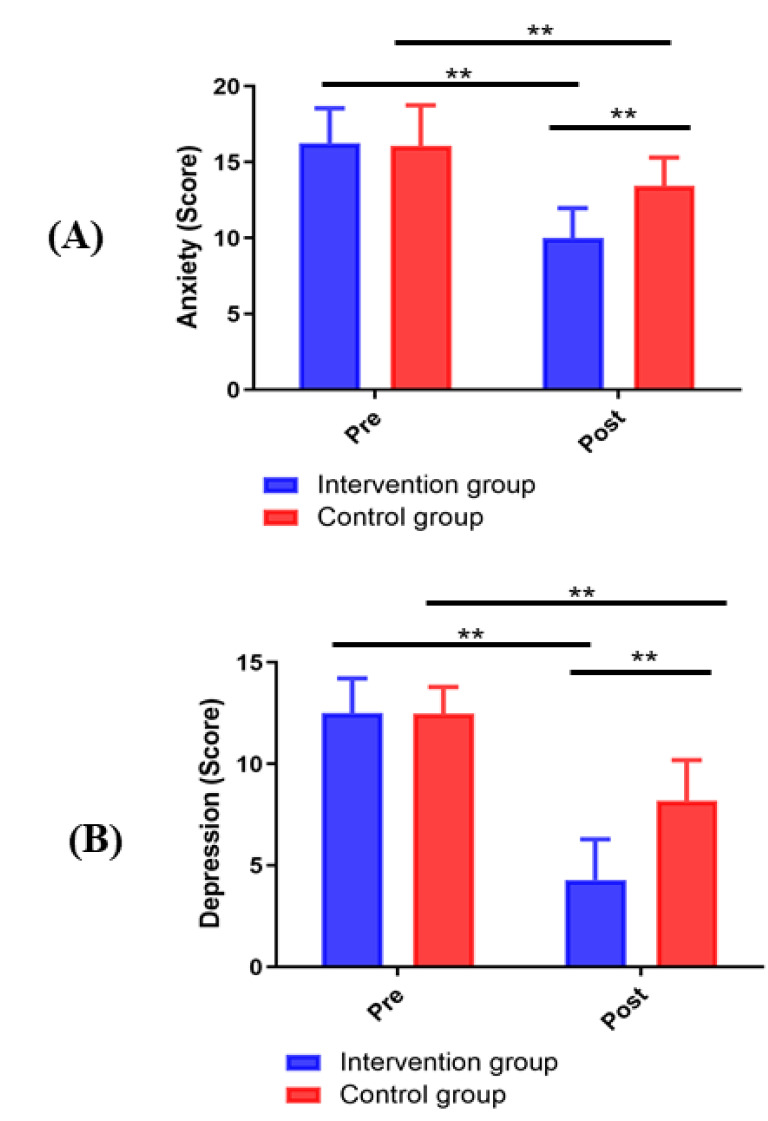
**Changes in HADS scores between groups**. (A) Anxiety. (B) Depression. **p < 0.01.

As shown in Fig. 7, no significant difference existed in the HADS score between the two groups before the intervention (*p *> 0.05). After the intervention, the two subscales of the HADS score (anxiety and depression) showed a statistically significant decrease in the intervention group compared with the control group (*p *< 0.01).

### 3.8 Comparison of Blood Glucose between the Two Groups

**Fig. 8.**
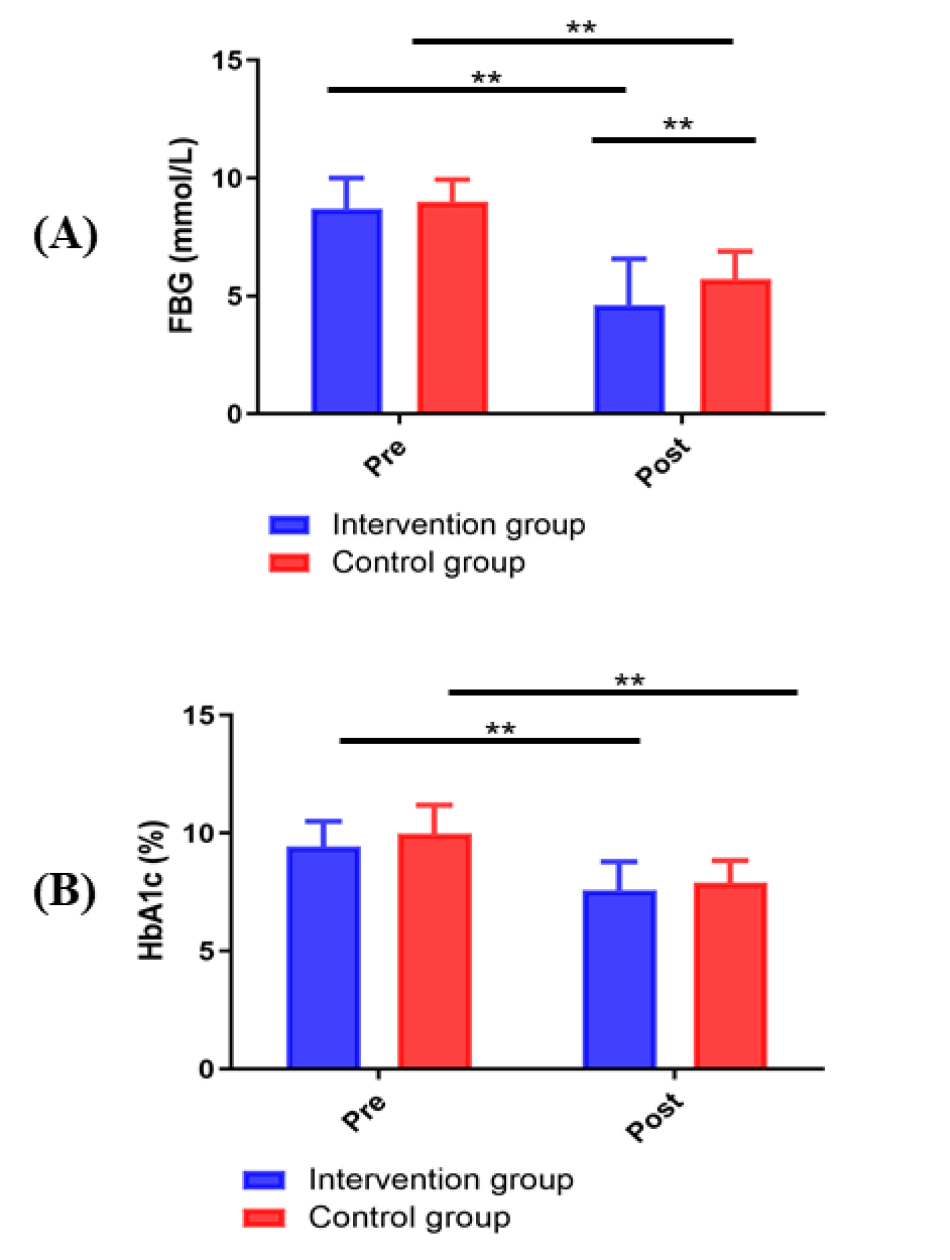
**Changes in FBG and HbA1c between groups**. (A) FBG. (B) HbA1c. **p < 0.01.

As shown in Fig. 8, before the intervention, no significant difference existed in FBG and HbA1c between the intervention group and control group (*p *> 0.05). After the intervention, compared with the control group, the intervention group showed a decrease in the serum levels of FBG (*p *< 0.01). However, no statistically significant difference existed between the intervention group and the control group in the serum levels of HbA1c (*p *> 0.05).

### 3.9 Comparison of Nursing Satisfaction between the Two Groups

**Fig. 9.**
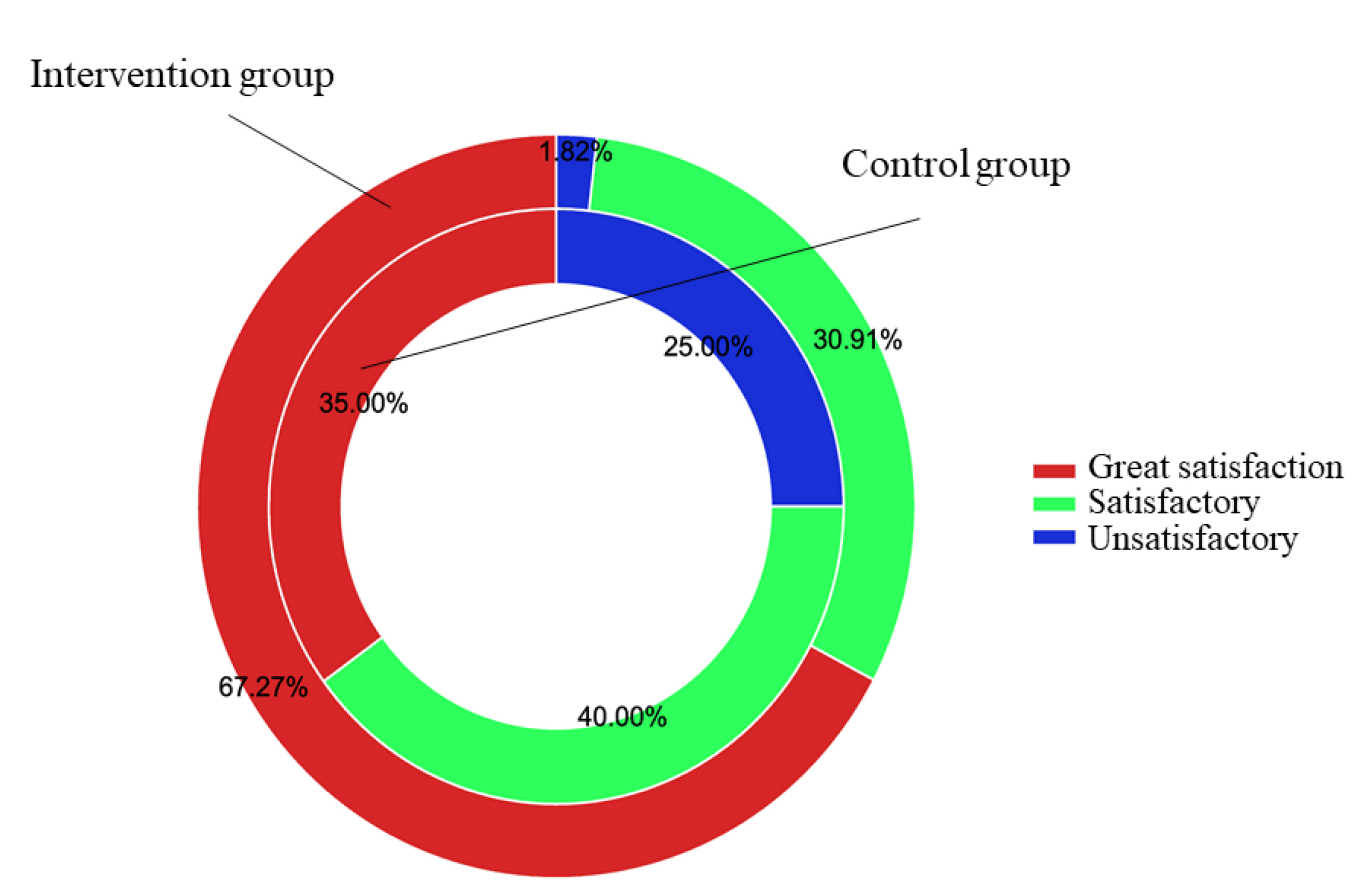
**Changes in nursing satisfaction between groups**.

In the intervention group, the proportions of participants reporting great satisfaction, satisfaction and unsatisfaction were 67.27%, 30.91% and 1.82%, respectively. In the control group, the proportions were 35%, 40% and 25%, respectively. A significant difference existed in nursing satisfaction between the two groups (98.18% vs. 75%; χ^2^ = 12.88, *p* = 0.01; Fig. 9).

## 4. Discussion

Overall, the six MIDAS subscales except for insecurity, and all dimensions of the CSMS, SRAHP, GSES and HADS scores, of the nurse-led individualised self-care group were significantly improved compared to those of the routine health education group (*p *< 0.05). Compared with the routine health education group (5.69 ± 1.43 mmol/L), the nurse-led individualised self-care group showed a decrease in the serum levels of fasting blood glucose (4.83 ± 1.57 mmol/L; *p *< 0.01). Thus, nurse-led individualised self-care might improve health-related quality of life, self-care ability, health behaviours, self-efficacy, social support and blood glucose among MI patients with type 2 diabetes.

**Fig. 10.**
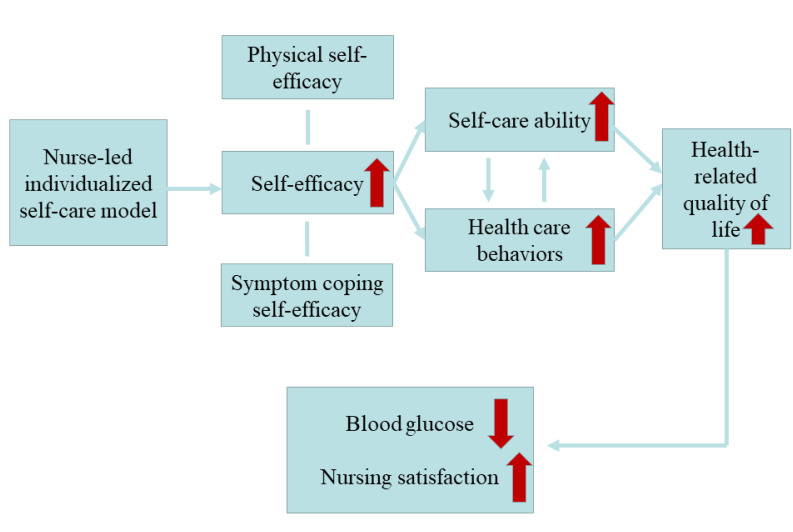
**The conceptual framework of the nurse-led individualised self-care model**.

Type 2 diabetes is caused by a relative lack of insulin secretion in the body, leading to metabolic disorders in the body. DM is also a high risk factor for cardiovascular disease. MI is a common complication in DM patients [32]. The nurse-led individualised self-care model not only has the advantages of traditional nursing models but also includes the multiple physiology and psychology needs of patients, which could achieve the goal of patient-centred nursing. The nurse-led individualised self-care model could combine the professional skills and long-term clinical experience of nursing specialists with the values and needs of patients, finally formulating a patient-centred nursing plan suitable for the individual disease situation. Therefore, the model could provide individualised and evidence-based clinical nursing services for MI patients with diabetes [33]. The conceptual framework of the model is shown in Fig. 10.

### 4.1 Assessment of Health-Related Quality of Life

Previously, Arnold *et al*. [34] reported an improvement trend for DM patients with heart disease in health-related quality of life after receiving a comprehensive nursing intervention, but no statistically significant difference existed between the two groups (*p *> 0.05). The results from Arnold *et al*. [34] are inconsistent with the results of our research. These differences may be associated with the larger sample size, more frequent follow-up, and increased family and social support in the intervention group. According to Wang *et al*. [35], at least three subscales of the MIDAS in the nursing intervention group must have better scores than the control group for the nursing intervention to be considered to have clinical value. In our research, after the nurse-led individualised self-care model intervention, compared with the baseline, the scores of six subscales (physical activity, emotional reaction, dependency, diet, concerns over mediation and side effects) of MI patients with diabetes were significantly lower than those of the control group, showing statistically clinical significance (*p *< 0.05). Therefore, the nurse-led individualised self-care model is a relatively novel nursing intervention model for MI patients with diabetes.

Interestingly, after the nurse-led individualised self-care model intervention, no significant change occurred in the score of the security subscale of MIDAS compared with the control group (*p *> 0.05). This may be related to the longer disease duration of DM (over 10 years). MI patients with diabetes have a longer disease duration and have strict scores for the insecurity dimension. Therefore, changing the insecurity subscale score of MIDAS may require a more intensive nursing intervention, longer follow-up and more sensitive measurement tools.

### 4.2 Assessment of Self-Care Ability, Health Care Behaviours and Self-Efficacy

Compared with the control group, the nurse-led individualised self-care model based on self-efficacy theory group showed that it could effectively improve the GSES self-efficacy score of MI patients with diabetes, improve their SRAHP score for healthy behaviour and finally improve their long-term self-management score level (*p *< 0.05). These findings are helpful to clarify the mechanism by which MI patients with diabetes have shown increased self-efficacy through self-management interventions [36]. The results of our research are somewhat in line with the previous study by Wu *et al*. [37]. Through multiple forms of health education, such as one-to-one, peer and online education, nurse-led individualised self-care models have helped patients establish healthy beliefs, establish healthy behaviours and enhance their self-confidence in managing chronic diseases. Thus, the model could improve the health status of patients and promote their functional recovery through the improvement of self-management behaviour and emotional control, ultimately achieving nursing satisfaction for MI patients with DM [38].

In line with the results of this study, Sasso *et al*. [39] reported that the adherence of patients with cardiovascular diseases to medication was the key issue that health care providers needed to pay attention to. Health care providers can do much to improve patient adherence by developing a trusting relationship, improving continuity of care and understanding the role of family support [39]. In our research, the medical adherence management dimension score in the intervention group was significantly higher than that in the control group (*p *< 0.05). Therefore, a strength of this study is the increased adherence to drug therapy. To explore this issue, the nurse-led individualised self-care model integrates resources such as hospitals, communities, patient family members and peer educators, forming a more systematic community-based transitional care intervention model. This not only can guide and supervise patient medicine-taking behaviour but also evokes patient intrinsic spontaneous medicine-taking behaviour, which ultimately improves adherence [40].

### 4.3 Assessment of Social Support and Psychological Status

In this research, the nurse-led individualised self-care model integrated family support and peer support. This family and peer-oriented intervention is particularly suitable for integration into Asian Confucian culture. Confucian culture in Asia emphasises the relationship between family and peers. For example, in daily life, family members often take the initiative of food preparation and diet management to take care of MI patients with diabetes. Peers encourage patients in their daily life, alleviate patients’ anxiety and depression, and play an important role in daily blood glucose monitoring and daily exercise. The results of this study suggest that compared with the control group, the social support SSRS scores in the intervention group significantly increased, whereas the HADS anxiety and depression scores significantly decreased (*p *< 0.05). The findings of this study are similar to those of Choi *et al*. [41], which suggested that family and peer support had a positive relationship with self-care behaviour improvement. Therefore, peer education and family support are effective ways to help MI patients with diabetes improve their self-management abilities and reduce anxiety and depression.

### 4.4 Assessment of Blood Glucose

Previous research has revealed that the reduction of FBG and HbA1c was closely associated with the improvement of patients’ self-efficacy and health-related quality of life [42]. The results of our research indicated that compared with the control group, the nurse-led individualised self-care model could significantly reduce the serum levels of FBG in MI patients with diabetes (*p *< 0.05). Welch *et al*. [43] suggested that the individualised self-care model could reduce the expression level of FBG in DM patients, which is in line with the results of our study. However, both usual nursing care and an individualised self-care model could reduce the expression level of HbA1c, but the expression of HbA1c between the two groups was not statistically significant (*p *> 0.05). The global guidelines for type 2 diabetes indicate that when HbA1c is below 7% (53 mmol/mol), it could minimise the risk of diabetes complications [44]. Therefore, the cardiovascular nursing specialists encouraged study participants to achieve and maintain the goal of an HbA1c level below 7% (53 mmol/mol). In this study, 65% of patients in the control group and 51% of patients in the intervention group had a higher HbA1c (>7%) at baseline. After the 12-week intervention, the average level of HbA1c in the control group and the intervention group was 7.6% and 7.9%, respectively. Therefore, the results of our research suggest that through the nurse-led individualised self-care intervention, the expression level of HbA1c in nearly half of the patients had reached the pre-set goal. Nurse-led individualised self-care could effectively prevent diabetic angiopathies and improve the health-related quality of life for MI patients with diabetes. However, no significant difference existed in the expression of HbA1c between the two groups (*p *> 0.05), inconsistent with the results of Steinsbekk *et al*. [45].

The average baseline value of HbA1c in Steinsbekk *et al*. [45] was 10.23%, which was higher than the baseline level of HbA1c in our study. Therefore, positive results were easier in the study of Steinsbekk *et al*. [45]. In addition, Steinsbekk *et al*. [45] had a long-term nursing intervention, which lasted around 12 months. In Steinsbekk *et al*.’s study [45], after the nursing intervention, HbA1c was significantly decreased by 4.4% at 6 months and 4.6% at 12 months. In our study, the difference between HbA1c at baseline and after the 12-week nurse-led individualised self-care intervention was 2.0%. This indicates that if the nursing intervention duration were extended to around 6 months and included more patients with MI, similar inter-group differences could be verified in our research.

### 4.5 Limitations

This clinical study may have several important limitations. First, the recruitment of participants from only one target medical centre could have contributed to some sampling bias, which may limit the generalisation of the results. Second, the majority of the outcomes in this study were self-reported, including health-related quality of life, self-care ability, health care behaviours, self-efficacy and social support, which can be susceptible to recall bias and inaccurate estimation. Third, due to the nature of the nursing interventions, blinding the participants and cardiovascular nursing specialists was not always feasible, which may have led to performance bias in this study. Finally, the duration of follow-up was short (only 3 months). No statistically significant differences existed between the intervention group and the control group in serum HbA1c levels (*p *> 0.05). However, Dobson *et al*. [46] found that the decrease in HbA1c at 24 months was significantly greater in the intervention group (self-management support program) compared to the control group (usual care; *p *< 0.05). Compared to this study, Dobson *et al*. [46] had a long follow-up duration of around 24 months. Therefore, if the follow-up duration were extended to around 24 months, similar differences between groups could be verified in our research.

## 5. Conclusions

Our pilot study provides preliminary evidence supporting the feasibility of implementing nurse-led individualised self-care, suggesting its preliminary effects in improving health-related quality of life, self-care ability, health behaviours, self-efficacy, social support and nursing satisfaction among MI patients with diabetes. However, considering the unblinded and pilot nature of this study, these positive results should be interpreted with caution. Future studies need to replicate this one in a larger population with a multi-centre design to verify the clinical significance of this intervention.

## Data Availability

The datasets used and/or analyzed during the current study are available from the corresponding author on reasonable request.

## Supplementary Material

Supplementary material associated with this article can be found, in the online version, at https://doi.org/10.31083/j.rcm2401031.
