# Supplementary material for: Effect of Nurse-Led Individualised Self-Care Model on Myocardial Infarction Patients with Diabetes: A Randomised Controlled Pilot Trial
Source: Rev Cardiovasc Med. 2023 Jan 16;24(1):31. doi: 10.31083/j.rcm2401031 (PMC11270464; doi:10.31083/j.rcm2401031)
Supplement: Supplementary file 1 [file 2153-8174-24-1-031-s1.zip › 2153-8174-24-1-031-s1.docx]

**Contents of health education**

| Contents of health education |
| --- |
| ☯ Overview of MI and DM: Briefly describe the correlation between MI and DM  ☯ Diet: Low sodium and carbohydrate diet; Standard sample menus for MI and DM  ☯Medication: Overview of medication usage for MI and DM; Individualized MI-DM medication principles, potential medicine conflicts, medical adherence to OTC and prescribed medication.  ☯ Symptom monitoring：Evaluate and explain chest pain, fatigue, insomina, depression and anxiety.  ☯ self-monitoring: Monitor weight, FBG and HbA1c; Relationship between MI-DM.  ☯Physical activity: duration, frequency, and safety of physical exercise  ☯ Oral and foot care |
